# Two-Dimensional Latent Space Manifold of Brain Connectomes Across the Spectrum of Clinical Cognitive Decline

**DOI:** 10.3390/bioengineering12080819

**Published:** 2025-07-29

**Authors:** Güneş Bayır, Demet Yüksel Dal, Emre Harı, Ulaş Ay, Hakan Gurvit, Alkan Kabakçıoğlu, Burak Acar

**Affiliations:** 1 VAVlab, Department of Electrical & Electronics Engineering, Boğaziçi University, Istanbul 34342, Türkiye; 2 Department of Electrical & Electronics Engineering, Fatih Sultan Mehmet Vakıf University, Istanbul 34015, Türkiye; dyukseldal@fsm.edu.tr; 3 Hulusi Behçet Life Sciences Research Laboratory, Neuroimaging Unit, Istanbul University, Istanbul 34093, Türkiye; emre.hari@istanbul.edu.tr (E.H.); psk.ulasay@gmail.com (U.A.); 4 Department of Physiology, Istanbul Faculty of Medicine, Istanbul University, Istanbul 34093, Türkiye; 5 Behavioral Neurology and Movement Disorders Unit, Department of Neurology, Faculty of Medicine, Istanbul University, Istanbul 34093, Türkiye; gurvit@istanbul.edu.tr; 6 Department of Physics, Koç University, Istanbul 34450, Türkiye; akabakcioglu@ku.edu.tr

**Keywords:** Alzheimer’s disease dementia, brain connectome, structural connectivity, graph neural networks, low-dimensional manifold, disease progression

## Abstract

Alzheimer’s Disease and Dementia (ADD) progresses along a continuum of cognitive decline, typically from Subjective Cognitive Impairment (SCI) to Mild Cognitive Impairment (MCI) and eventually to dementia. While many studies have focused on classifying these clinical stages, fewer have examined whether brain connectomes encode this continuum in a low-dimensional, interpretable form. Motivated by the hypothesis that structural brain connectomes undergo complex yet compact changes across cognitive decline, we propose a Graph Neural Network (GNN)-based framework that embeds these connectomes into a two-dimensional manifold to capture the evolving patterns of structural connectivity associated with cognitive deterioration. Using attention-based graph aggregation and Principal Component Analysis (PCA), we find that MCI subjects consistently occupy an intermediate position between SCI and ADD, and that the observed transitions align with known clinical biomarkers of ADD pathology. This hypothesis-driven analysis is further supported by the model’s robust separation performance, with ROC-AUC scores of 0.93 for ADD vs. SCI and 0.81 for ADD vs. MCI. These findings offer an interpretable and neurologically grounded representation of dementia progression, emphasizing structural connectome alterations as potential markers of cognitive decline.

## 1. Introduction

Alzheimer’s Disease and Dementia (ADD) is a progressive neurodegenerative disorder characterized by the gradual decline of behavioral and cognitive functions. It often manifests in the form of deteriorating reasoning, diminished memory, reduced attentional capacity, and weakened executive function. Though commonly associated with aging, ADD reflects a distinct pathological process that begins years before clinical symptoms emerge [1]. Early in this continuum, individuals may experience Subjective Cognitive Impairment (SCI)—self-perceived cognitive decline without measurable deficits—often preceding Mild Cognitive Impairment (MCI), where objective impairments are detectable but daily functioning remains largely intact. MCI, particularly its amnestic form, is considered a high-risk state for progression to ADD dementia. Understanding these early stages is vital for timely diagnosis and intervention, as the disease advances silently and irreversibly over time.

Brain networks (connectomes) offer a useful framework for characterizing the organization of the brain and examining the alterations associated with neurological disorders. They are typically modeled as graphs, where nodes represent anatomically defined cortical regions—often delineated using standardized brain atlases [2]—and edges encode the relationships or connections between them. Connectomes are commonly categorized into two principal types: structural networks (sNets) and functional networks (fNets). Structural networks are derived from white matter pathways, estimated through diffusion-based imaging techniques, and reflect the physical connectivity between regions. In contrast, functional networks are constructed by quantifying statistical dependencies—such as correlations—between Blood-Oxygen-Level Dependent (BOLD) signals recorded from distinct cortical areas. Both structural and functional connectomes provide complementary insights and have been extensively utilized in Alzheimer’s Disease and Dementia (ADD) research. They offer valuable perspectives on the neural disconnection patterns that are characteristic of the disorder [3,4].

In the context of Alzheimer’s Disease and Dementia (ADD) research, the majority of studies have predominantly focused on functional brain networks (fNets), while investigations utilizing structural brain networks (sNets) remain relatively limited. Nonetheless, both network types offer complementary insights and have demonstrated utility in elucidating the connectome alterations associated with the disease. A range of graph-theoretical analyses have been employed in the literature, often examining canonical metrics such as small-worldness, hub topology, clustering coefficient, and community structures [5,6,7,8]. Beyond these conventional approaches, various computational methods have also been explored to model and interpret network-level changes in ADD. These include convolutional neural networks (CNNs) [9], frequency-based approaches [10], and more recent techniques such as tensor factorization [11,12]. However, each of these methods present with limitations. Traditional graph metrics often yield shallow insights, while many advanced techniques are not inherently designed for graph-structured data.

Graph Neural Networks (GNNs) have recently gained significant traction across various real-world domains, including biomedical applications [13,14,15]. Given their inherent ability to model graph-structured data, GNNs are particularly well suited for extracting meaningful features from brain connectomes as well. They have been applied to tasks such as gender classification [16], autism diagnosis [17], emotion recognition [18], brain activity decoding [19], auditory attention detection [20], and neuropsychiatric disorder classification [21].

In the context of Alzheimer’s Disease and Dementia (ADD), the integration of Graph Neural Networks (GNNs) with brain connectomes remains an evolving area of research. One study proposes a hybrid RNN-GCN model for classifying patients with Mild Cognitive Impairment (MCI) [22]. Another utilizes connectomes derived from denoised T1-weighted MRI to distinguish among MCI, Early MCI (EMCI), Alzheimer’s Disease and Dementia (ADD), and healthy controls [23]. A different approach leverages functional networks and temporal features extracted from BOLD signals to support early diagnostic tasks [24]. Collectively, these studies highlight the potential of GNNs for modeling complex brain network alterations associated with ADD.

However, the majority of existing research has primarily focused on classification tasks. In contrast, the conceptualization of the cognitive decline spectrum remains a critical yet underexplored domain. Moreover, efforts to characterize this spectrum with compact representations have received even less attention. Low-dimensional representations have been widely explored across various biomedical applications [25,26,27]. In the context of brain data, they have been utilized for diverse applications, such as classification using low-dimensional embeddings of MRI data [28], graph embeddings in discriminative analyses of ADD patients [29], and studies exploring structure-function associations in brain networks [30]. One recent study introduces a discrepancy analysis framework coupled with GNNs to examine progression across SCI, MCI, and ADD populations [31]. This approach aims to capture transitional patterns in cognitive decline rather than discrete diagnostic categories but it lacks full characterization of a disease manifold. Ref. [32] develops a prognostic framework using functional connectome manifolds derived from functional gradients, combined with survival analysis to estimate conversion risks to ADD; however, the focus remains limited to predicting individual transitions towards the end of the ADD spectrum. Ref. [33] projects functional networks onto a continuous gradient space to quantify dispersion correlated with cognitive scores, though this gradient space does not model disease trajectories. Ref. [34] applies autoencoders to extract low-dimensional embeddings from functional connectivity matrices to identify ADD subtypes, yet the study concentrates on subtype separation rather than progressive stages. Ref. [35] employs UMAP on cortical thickness data to investigate MCI-to-ADD transitions, but excludes connectomic features and lacks regional specificity. Ref. [36] models neurodegeneration using imaging and metabolic data to relate anatomical variation to various dementia types, yet does not address the full progression from the early stages to the late stages specific to ADD. Ref. [37] constructs a UMAP-based manifold using high-dimensional clinical, neuropsychological, genetic, and biomarker data across multiple timepoints to reveal patterns of heterogeneity and progression in ADD; however, the absence of neuroimaging data precludes interpretation in terms of underlying brain network dynamics or regional involvement. Ref. [38] applies locally linear embedding (LLE) to MRI data to visualize progression over time, yet the resulting representation offers limited interpretability and lacks separation between intermediate stages.

Collectively, these studies highlight the promise of low-dimensional modeling for analyzing neurological data, but most are constrained by limited scope—focusing on classification, subtype delineation, or visualization—while falling short of capturing the full continuum of cognitive decline, constructing an interpretable brain-based disease manifold, or providing an in-depth analysis of it.

### 1.1. Motivation and Objectives

The central motivation of this study is the hypothesis that, despite the high dimensionality of brain connectome data, clinical cognitive decline is driven by a relatively small number of structural changes. These changes are expected to be spatially complex but constrained within a low-dimensional manifold. Demonstrating this could deepen our understanding of disease progression and provide a foundation for future diagnostic tools.

The objective of this work is to test the hypothesis that structural brain connectomes, across the full cognitive decline spectrum, lie on a low-dimensional, interpretable manifold. We designed a GNN-based deep learning pipeline that produces embeddings of brain networks. These embeddings are then projected onto a two-dimensional space, enabling visualization and analysis of the manifold. Unlike many prior works focused on discrete classification, our primary goal is not to achieve high diagnostic performance; rather, our primary goal is to provide a data-driven, structural characterization of dementia progression. The primary contributions of this paper can be summarized as follows:

- We propose a GNN-based deep learning framework that reveals a two-dimensional manifold of brain connectomes, capturing the continuum of clinical cognitive decline.
- We show that the learned manifold aligns with established clinical and anatomical patterns of dementia, offering an interpretable and neurologically grounded representation of disease progression.
- We find that the low-dimensional structure of cognitive decline reflects complex yet consistent alterations across the clinical cognitive decline spectrum.

### 1.2. Paper Structure

The remainder of the paper is organized as follows. Section 2 describes the materials and methods, including the mathematical formulation and architectural pieces. Section 3 explains the proposed framework in detail and how it is trained. Section 4 presents the learned manifold structure and reports our experimental findings, including results from an ablation study. Section 5 interprets these findings in the context of diagnostic staging and structural connectivity. Finally, Section 6 offers concluding remarks and outlines potential directions for future research.

## 2. Materials and Methods

### 2.1. Brain Networks

A weighted, undirected, loop-free graph can be defined with its vertex/node set, $V=\{v_{i}\}$ s.t. |V|=N, edge set, $E=\{e_{ij}\}$, and adjacency matrix, $A_{N\times N}=\left\{a_{ij}\right\}$, where $a_{ij}=a_{ji}\wedge a_{ii}=0$ and $a_{ij}=0$ if $e_{ij}\notin E$. The nodes/vertices can have associated features, $x_{i}$, and the graph’s feature matrix can be constructed as $X_{N\times F}$, where *F* is the dimension of the feature. Therefore, the graph data with all the additional information can be fully defined as G=(V,E,A,X).

Brain networks can be represented as graphs. Vertices/nodes represent cortical regions, and the edges represent different associations between those regions, based on the functional or structural connectivity definitions of the network generation process. Nodes and edges can have associated features/embeddings that encode node/edge specific information. In this study, we are going to focus on only node features.

### 2.2. Dataset

T1-weighted MRI data were acquired using a 3D FFE (Fast Field Echo) pulse sequence with multi-shot TFE (Turbo Field Echo) imaging mode. The acquisition parameters were TE/TR=3.8 ms/8.3 ms, $\mathrm{flip}\;\mathrm{angle}=8^{\circ },\mathrm{FOV}=220\times 240\;{\mathrm{mm}}^{2}$, $1.0\;{\mathrm{mm}}^{3}$ isotropic voxels, and 180 slices. Diffusion-weighted imaging (DWI) data were acquired with 120 gradient directions with a maximum gradient strength of 40mT/m and a high slew rate of 200mT/m/ms, at 6 shells in the q-space. FOV was $220\times 236\;{\mathrm{mm}}^{2}$ with imaging matrix 112×112 and 71 slices with slice thickness of 2.27mm. rs-fMRI data were acquired using the Fast Field Echo (FFE) technique in a multi-slice mode with a single-shot EPI sequence. The acquisition parameters were as follows: $\mathrm{TE}/\mathrm{TR}=30\;ms/3000\;ms,\mathrm{flip}\;\mathrm{angle}=80^{\circ }$, $\mathrm{FOV}=212\times 199\;{\mathrm{mm}}^{2}$. A total of 200 EPI volumes and 48 axial slices were acquired with an eyes-closed approach.

Written informed consent was obtained from 88 participants in accordance with institutional ethics committee approvals (Istanbul University, Istanbul Faculty of Medicine, Ethics Committee Approval: 877/30.05.2014; Bogazici University, Ethics Committee Approval: 2014-1/17.02.2014). The study cohort consisted of three groups: one group comprised individuals with early-stage Alzheimer’s Disease and Dementia (ADD, n = 18); the second group comprised individuals with amnestic Mild Cognitive Impairment (MCI, n = 46); the third group comprised individuals with Subjective Cognitive Impairment (SCI, n = 24). Patients in the ADD group met the clinical diagnostic criteria for typical ADD of the National Institute on Aging and Alzheimer’s Association (NIA-AA) and had a CDR score of 0.5 or 1, corresponding to mild dementia [39]. The MCI group consisted of patients with a total free recall (TFR) score ≤ 27 or a cue index (CI) of less than 0.67 on the Free and Cued Selective Recall Test (FCSRT) based on Petersen diagnostic criteria, and a CDR–Sum of Boxes (CDR-SOB) score of 0.5 or 1 when the CDR score was 0.5 [40,41]. Finally, the SCI group consisted of participants with subjective memory complaints who had an FCSRT-TFR score greater than 27 or an CI greater than 0.67 and a CDR score of 0 [42]. SCI participants were recruited through community advertisements and were required to score at least 1 on either the Cognitive Functions Instrument Subject form (CFI-S) or the Cognitive Functions Instrument Study Partner form (CFI-SP). Final diagnoses were confirmed through comprehensive neurological and neuropsychological assessments, supported by cranial magnetic resonance imaging (MRI) and reviewed by a panel of behavioral neurologists. Exclusion criteria encompassed a history of neurological or psychiatric conditions affecting cognition, alcohol or substance abuse, significant head trauma with loss of consciousness, and the presence of moderate–severe white matter hyperintensities (Fazekas score 2–3) on MRI. Individuals with contraindications for MRI scanning were also excluded from participation.

### 2.3. Brain Connectome Construction

A multi-modal preprocessing pipeline was employed to generate co-registered three-dimensional neuroimaging datasets comprising T1-weighted structural MRI and diffusion-weighted imaging (DWI) volumes. The pipeline was implemented using established neuroimaging toolkits, including FSL 5.0, FreeSurfer 5.3, and TORTOISE 2.5.1 (https://vavlab.bogazici.edu.tr/brainet-structural-and-functional-brain-network-analysis, accessed on 16 July 2025). Diffusion tensor estimation was performed on multi-shell DWI acquisitions using a dual tensor basis solution to the Stejskal–Tanner equations, providing robust modeling of water diffusion patterns in white matter tissue. Deterministic tractography was performed using a fourth-order Runge–Kutta (RK4) integrator with a minimum fractional anisotropy (FA) of 0.15, 0.7 mm step size (≈half the voxel size), 20 mm minimum streamline length, and 35° curvature threshold [43]. Bidirectional tracking was initiated 30 times per white matter voxel with FA≥0.15. sNET nodes were defined based on the Destrieux anatomical atlas, which parcellates the cortical surface into anatomically distinct regions [44]. sNET edge weights were defined by the total number of streamlines connecting each cortical parcel pair. To enhance anatomical precision, streamline endpoints were probabilistically associated with cortical parcels (connectome nodes) using a 3D Gaussian kernel density function, which is derived from the local diffusion profile at the streamline endpoint, thereby modeling the spatial uncertainty of fiber tips relative to the parcellation boundaries. This formulation follows the methodology outlined in [11], and the standard deviations of the Gaussian kernels were computed using the procedure detailed in [45]. Connectivity values between nodes were subsequently normalized by the combined volumes of the connected parcels to account for inter-regional size variability, yielding weighted, undirected structural networks for further analysis.

### 2.4. Graph Neural Networks

Graph Neural Networks (GNNs) are the natural choice for processing and learning from graph data. Most modern GNNs follow a message passing strategy, where nodal features are updated by aggregating information from their neighborhood [46]. For a vertex, *v*, this operation can be written in the following generalized form:

(1) $$a_{v}=\mathrm{Aggregate}\left(\left\{x_{u}\;:u\in N\left(v\right)\right\}\right),\;\;\;\;x_{v}^{new}=\mathrm{Update}\left(x_{v},a_{v}\right),$$

where N(v) denotes the neighboring nodes of the vertex *v*, and $x_{v}$ is the nodal feature vector for *v*.

The above generic aggregate/update procedure describes how the information propagates throughout the graph within one iteration. Various types of GNNs differ in the way they define these aggregate and update steps. A very common type of GNN, Graph Convolutional Networks (GCNs) [47], use weighted sum aggregation, where the weights are defined by the spectral interpretations of the graph topology. Graph Isomorphism Networks (GINs) use sum aggregation [48]. Graph Attention Networks (GATs) and Graph Transformers calculate an attention matrix based on nodal features for weighted sum aggregation [49,50]. GraphSAGE uses max operator for aggregation [51]. Some GNNs also incorporate edge features into the aggregation process [52].

GIN’s aggregate and update steps can be explicitly described as follows:

(2) $$x_{v}^{new}=\phi \left(\left(1+\epsilon \right)x_{v}+\sum_{u\in N(v)}x_{u}\right),$$

where ϕ is a multi-layer perceptron (MLP) and ϵ is an irrational number. The choice of the update function is based on the Universal Approximation Theorem [53] and increases the predictive power [48].

In GAT, aggregate and update steps can be explicitly described as follows:

(3) $$x_{v}^{new}=\sum_{u\in N(v)}\alpha_{u,v},z_{u},$$

where $z_{u}=Wx_{u}$ is the linear transformed feature vector of vertex *u*, $\alpha_{u,v}$ is the attention matrix entries for neighboring vertices *u* and *v*, defined as

(4) $$\alpha_{u,v}=\frac{\exp(h(a^{T}(z_{u}\left|\right|z_{v})))}{\sum_{a\in \{N(v)\cup v\}}\exp(h(a^{T}(z_{a}\left|\right|z_{v})))},$$

where *h* is LeakyRELU activation, a is called the attention kernel and is a learnable layer parameter twice the size of the transformed feature dimension. For non-neighbor vertices, the corresponding attention matrix entry is 0.

To make decisions at the graph level, such as graph classification, it is necessary to aggregate node-level features into a comprehensive graph-level representation. Common approaches include global pooling strategies such as mean, sum, or max pooling. More advanced methods have also been proposed, including SortPooling [54], which applies a two-stage sorting algorithm to node features and node indices, and Set2Set [55], which employs a Long Short-Term Memory (LSTM) network [56] to capture complex dependencies among node features. In this study, we adopt a specialized variant of the attention-based pooling mechanism proposed by [57]. Following [57], the attention-based graph-level readout is computed as:

(5) $$x_{G}=\sum_{\forall vs.\in V}\mathrm{softmax}\left(\phi_{\mathrm{gate}}\left(x_{v}\right)\right)⊙x_{v},$$

where $x_{G}$ denotes the summarized graph feature vector, and $\phi_{\mathrm{gate}}:R^{F}\to R$ is a learnable neural network applied to each node’s feature vector.

### 2.5. AI-Assisted Editing

The manuscript was reviewed with the help of OpenAI’s GPT-4o model, focusing on improving grammar and writing style to enhance the overall clarity and readability.

## 3. Proposed Framework: Attention-Guided Graph Embedding and Manifold Projection

**Figure 1:**
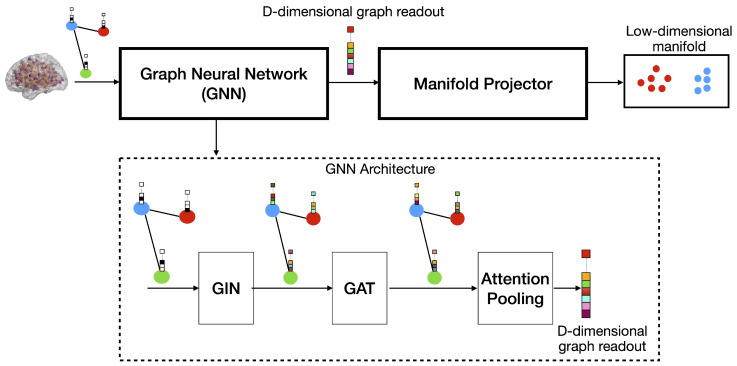
Overview of the proposed framework comprising two primary stages: a Graph Neural Network (GNN) for feature extraction and a manifold projector for dimensionality reduction. The GNN module sequentially integrates a Graph Isomorphism Network (GIN), a Graph Attention Network (GAT), and an attention-based pooling mechanism to encode the brain connectivity graph into a D-dimensional feature vector. This high-dimensional embedding is then passed to the manifold projector, where it is transformed into a low-dimensional representation using Principal Component Analysis (PCA) for visualization or downstream analysis.

Using the building blocks described in Section 2.4, we construct a low-dimensional manifold learning framework, as illustrated in Figure 1. The model consists of two primary stages: a Graph Neural Network (GNN) for graph embedding computation and a manifold projector for dimensionality reduction. The input to the network comprises brain networks with binarized edges and one-hot encoded node feature vectors representing node indices. Edge binarization is performed by checking for the presence of fibers between cortical regions.

The GNN module is composed sequentially of a Graph Isomorphism Network (GIN), a Graph Attention Network (GAT), and an attention-based pooling layer. GIN is employed for its high expressive power in capturing complex graph topologies, enabling a rich encoding of brain connectivity patterns. GAT further refines these representations by adaptively weighting node interactions, thereby allowing the model to focus on the most informative brain connections. Finally, an attention-based pooling mechanism is used to aggregate the most relevant node features into a compact graph-level representation. These resulting embeddings are well-suited for subsequent low-dimensional manifold analyses, facilitating the exploration of structural differences among SCI, MCI, and ADD populations.

The resulting D-dimensional graph embeddings are then projected onto a low-dimensional space using Principal Component Analysis (PCA). PCA is selected for its interpretability, robustness, ease of reconstruction, and its ability to project new, unseen samples without requiring retraining. Its linear nature allows approximate inversion, making it particularly suitable when the reconstruction or traceability of the original high-dimensional features is desired. The resulting low-dimensional manifold supports intuitive visualization and facilitates the analyses of structural patterns among SCI, MCI, and ADD populations.

To learn the weights of the Graph Neural Network (GNN) block, it is temporarily coupled with a multi-layer perceptron (MLP) to form an end-to-end supervised pipeline. This composite model is trained on a binary classification task (e.g., ADD vs. SCI), which provides a supervisory signal for optimizing GNN parameters. The MLP receives the D-dimensional graph-level embeddings generated by the GNN and produces class probabilities through a softmax activation. It comprises two fully connected layers with nonlinear activation functions. During training, the GNN and MLP are jointly optimized using labeled data, enabling the GNN to learn representations that are both structurally meaningful and discriminative with respect to disease status. After training, the MLP is discarded and the GNN is repurposed as a standalone embedding module for constructing the low-dimensional manifold.

After the GNN + MLP pipeline is fully trained, Principal Component Analysis (PCA) is fit to the D-dimensional graph embeddings derived from the training set. This process identifies the principal components that capture the dominant axes of variance within the learned representation space. The resulting PCA transformation is then fixed and subsequently applied to project new, unseen samples onto the same low-dimensional manifold during evaluation. This strategy ensures consistency between training and inference, while eliminating the need to retrain or update the projection step. By decoupling manifold construction from the GNN’s supervised training objective, the framework remains modular, interpretable, and amenable to reproducible analysis across subject groups.

## 4. Results

### 4.1. Low-Dimensional Manifold of Brain Connectomes

We trained the model architecture illustrated in Figure 1 to address the binary classification task of distinguishing Alzheimer’s Disease and Dementia (ADD) from Subjective Cognitive Impairment (SCI). Model optimization was performed using the Adam optimizer [58], with both the learning rate and weight decay set to 0.001, over the course of 100 training epochs. The GIN layer outputs 128-dimensional feature representations, while the GAT layer produces 64 dimensional outputs. The MLP component included a hidden layer of 32 neurons and an output layer configured to align with the binary cross-entropy loss used during training. All architectural and training hyperparameters were selected empirically. After training, all available ADD and SCI samples were passed through the network, and 64 dimensional graph embeddings were extracted from the output of the Graph Neural Network (GNN) block. Principal Component Analysis (PCA) was then applied, yielding a 2D manifold that captured 99% of the total variation in the dataset.

**Figure 2:**
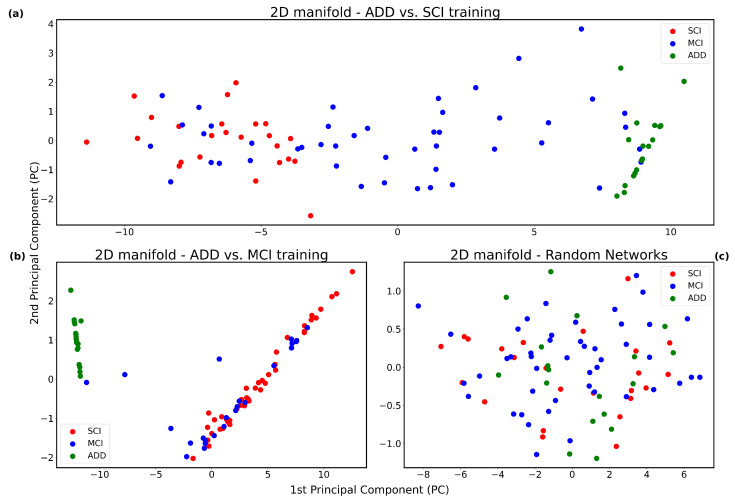
(**a**) The model described in Figure 1 is trained on the classification task of Alzheimer’s Disease and Dementia (ADD) versus Subjective Cognitive Impairment (SCI). Principal Component Analysis (PCA) is applied to obtain a two-dimensional representation. Mild Cognitive Impairment (MCI) samples are then projected onto this representation. Notably, MCI instances predominantly occupy the intermediary space between ADD and SCI clusters, which aligns with their intermediate position along the cognitive decline continuum. (**b**) The same experimental procedure is conducted using a model trained to discriminate between ADD and MCI. Here, SCI samples are projected onto the learned manifold. In contrast to (**a**), SCI instances are interspersed with MCI samples rather than forming a distinct cluster between the two classes. This observation is consistent with the proximity of SCI and MCI on the cognitive spectrum. (**c**) As a control experiment, we repeat the ADD vs. SCI classification using randomized brain networks that preserve the original data’s sparsity characteristics. When MCI samples are projected onto the corresponding PCA space, all three classes appear highly intermingled. The absence of distinct clustering in this setting supports the notion that the observed separability in (**a**,**b**) is not due to random chance but reflects genuine structural patterns in the brain network data.

Following this, we projected the graph embeddings of all MCI samples (which were excluded in the classification model training) onto the two-dimensional manifold, yielding the visualization depicted in Figure 2a. An analogous experiment was conducted for the ADD versus MCI classification task, where the SCI cases were excluded from the classification model training and later projected on the learned manifold. The resulting manifold is presented in Figure 2b. Note that the SCI cases are interleaved with MCI cases, which is the closest group to SCIs across the spectrum of clinical dementia.

To evaluate the statistical significance of the observed patterns, the experiment was repeated using randomly generated brain networks with sparsity levels matched to those in the original dataset, with the corresponding manifold shown in Figure 2c. The choice of two-dimensional projection was justified as it preserved 99% of the variance in the original embedding space, whereas for random brain networks, the intrinsic dimensionality required to retain comparable variance was determined to be at least four-dimensional.

**Figure 3:**
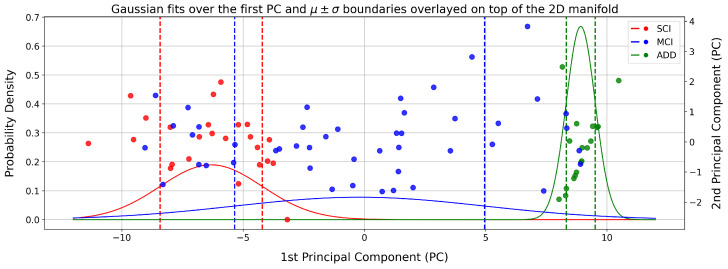
The two-dimensional manifold shown in Figure 2 was obtained by applying PCA to the GNN-derived graph embeddings in Figure 1. A univariate Gaussian distribution is then fitted to the first principal component of each diagnostic group. The resulting probability density functions are shown along with vertical dashed lines marking the μ±σ boundaries. To visualize how these probabilistic boundaries align with the full manifold, the two-dimensional PCA projection is overlaid as a scatter plot on a secondary y-axis.

Building upon the visualization in Figure 2a, we partitioned the manifold into regions where each clinical group is statistically concentrated. These regions were derived by fitting univariate Gaussian distributions to the first principal component (PC1) and defining cohort boundaries based on one standard deviation from the mean, as shown in Figure 3. Subsequently, individuals falling within these statistically defined intervals were aggregated to form distinct cohorts. These cohorts serve as the basis for the downstream analyses and discussions elaborated in Section 5.

### 4.2. Classifying Different Stages of Dementia

The model architecture depicted in Figure 1 was employed to extract low-dimensional manifolds representing the continuum of disease progression. This section demonstrates that the derived manifolds are outputs of a model exhibiting robust predictive capabilities. For the three binary classification tasks—namely, Alzheimer’s Disease and Dementia versus Subjective Cognitive Impairment (ADD/SCI), Alzheimer’s Disease and Dementia versus Mild Cognitive Impairment (ADD/MCI), and Mild Cognitive Impairment versus Subjective Cognitive Impairment (MCI/SCI)—the classification performance is summarized in Table 1. The area under the receiver operating characteristic curve (ROC-AUC) is reported as the primary evaluation metric, as it is well-suited for comparisons in the presence of class imbalance, which varies across the three classification tasks. It also obviates the need to select a specific decision threshold, thereby providing a threshold-independent assessment of model performance. In contrast, conventional accuracy measures would be less informative under these circumstances.

Given the limited sample size of the dataset, performance estimates were obtained using leave-one-out cross-validation (LOOCV) and results were aggregated over ten independent runs to ensure robustness. The model achieves high discriminative performance for distinguishing ADD from both MCI and SCI, whereas its ability to differentiate between MCI and SCI is notably weaker. Consequently, the manifolds generated from the ADD/SCI and ADD/MCI classification tasks are underpinned by models with demonstrably high predictive power, thereby lending greater validity to the structural insights gleaned from their low-dimensional representations.

### 4.3. Ablation Study

Subsequently, we conducted an ablation study to evaluate the contribution of individual model components. By selectively removing or modifying specific sections of the architecture, we assessed their impact on overall performance. The analysis was carried out for both the ADD/SCI and ADD/MCI classification tasks. The MCI/SCI task was excluded due to the model’s insufficient predictive performance, rendering further ablation analysis uninformative. The evaluation focused on key components of the GNN block. Specifically, the following architectural interventions were explored:

- Substituting the attention-based graph readout mechanism with a simpler aggregation function, such as sum-pooling;
- Removing the graph attention (GAT) layer entirely;
- Replacing the Graph Isomorphism Network (GIN) layer with a Graph Convolutional Network (GCN);
- Eliminating the GIN layer altogether.

The results of the ablation study are comprehensively reported in Table 2.

## 5. Discussion

### 5.1. Cohort Composition and Methodological Considerations

While the dataset used in this study is modest in size, it is notably homogeneous, with all samples acquired under identical imaging protocols and processing conditions. This deliberate design choice was made to minimize variability arising from acquisition differences, which can confound analysis and obscure meaningful patterns in larger, more heterogeneous datasets. The uniformity of the cohort provides a controlled setting in which to study structural brain changes across the cognitive decline continuum. This, in turn, allows for more confident interpretation of the learned representations.

The dataset also exhibits a degree of class imbalance. However, the largest diagnostic group, MCI, was excluded from the training phase, as the model was specifically designed to learn from the two clinical endpoints—SCI and ADD—in order to construct a well-defined disease trajectory. These two groups exhibit a class ratio of 3:4, which provides a fair balance for training and supports the stability and interpretability of the learned manifold representations.

### 5.2. Architectural Insights

As evidenced by the findings in Table 2, the Graph Isomorphism Network (GIN) layer emerges as the most critical component influencing model performance. Substituting the GIN layer with a Graph Convolutional Network (GCN) or entirely omitting it leads to a marked decline in predictive accuracy, underscoring its pivotal role. This difference can be attributed to the GIN’s superior expressive capacity, which is provably stronger than that of GCNs. While GCNs rely on mean-based aggregation, GIN matches the discriminative power of the Weisfeiler–Lehman (WL) graph isomorphism test [48]. GCNs are also more prone to representational homogenization, even at shallow depths, due to their inherent averaging operations that promote early-stage smoothing of node features [59,60]. In addition, they are constrained by the underlying graph topology. GCNs rely on fixed neighborhood structures for message passing. This can limit their ability to capture salient or global subgraph features relevant to the classification task. This limitation becomes particularly pronounced when the GCN layer precedes a Graph Attention Network (GAT) layer, as oversmoothed or topologically constrained inputs reduce the effectiveness of attention mechanisms. In contrast, the GIN’s ability to retain fine-grained structural distinctions enables the GAT layer to operate on more informative embeddings. Furthermore, incorporating attention mechanisms in later stages—particularly during the graph readout phase—yields additional performance gains. This improvement is likely due to the attention mechanism’s ability to emphasize task-relevant substructures [49].

### 5.3. Two-Dimensional Manifold Structure

In the ADD versus SCI classification task, the model was trained utilizing all available ADD and SCI samples. Intermediate graph embeddings were extracted from the output of the attention-based readout layer and subsequently projected into a two-dimensional space via Principal Component Analysis (PCA), as described in Section 3. Following model training, MCI samples were input into the network, and their resulting graph embeddings were similarly reduced in dimensionality and overlaid on the existing manifold, as illustrated in Figure 2a.

The resulting two-dimensional manifold reveals that MCI samples predominantly occupy the intermediate region between the ADD and SCI clusters, suggesting a continuum of disease progression. Conceptually, this intermediate positioning is consistent with the clinical trajectory of cognitive decline, as MCI represents a transitional phase between SCI and ADD, despite its known heterogeneity. To further investigate this hypothesis, we conducted additional validation experiments.

Specifically, the model was retrained for the ADD versus MCI classification task, after which SCI samples were introduced. The resulting manifold, depicted in Figure 2b, demonstrates a clear separation between ADD and MCI clusters, with SCI samples predominantly aligning with the MCI group. This observation supports the view that SCI subjects are cognitively and structurally closer to MCI patients, aligning with the broader understanding of disease progression.

To assess whether the observed manifold structure reflects meaningful brain network organization rather than random variation, we conducted a control experiment in which the model was trained entirely on randomly generated brain networks with similar sparsity characteristics. A separate set of random networks was then introduced as the third class and projected onto the resulting manifold. Unlike the structured separability observed in models trained on real data, the manifold derived from random networks did not exhibit discernible class separation. Instead, all three classes were largely superimposed (Figure 2c). This result suggests that the manifold structure observed in our main experiments is not an artifact of the model architecture or dimensionality reduction technique, but is instead driven by the intrinsic connectivity patterns present in actual brain data.

Additionally, to statistically analyze class separability in the full manifold shown in Figure 2a, we performed a permutation test based on the ROC-AUC of a multi-class logistic regression classifier trained on the 2D coordinates. The classifier achieved a ROC-AUC of 0.91, which we compared against a null distribution obtained by repeatedly shuffling the labels. The resulting *p*-value was <$10^{-4}$, indicating that the observed separability is highly unlikely to occur by chance and further supports the statistical robustness of the learned representation.

Collectively, these findings suggest that the intermediate positioning of MCI subjects between ADD and SCI is not an artifact of random variation but rather reflects an underlying disease progression trajectory embedded within the manifold structure.

Furthermore, we assessed the dimensionality of the learned manifold by examining the variance explained by the principal components. For real brain network embeddings, approximately 99% of the variance was captured within the first two principal components. This indicates that a two-dimensional projection effectively preserves the essential structural information. In contrast, embeddings derived from random networks required at least four dimensions to achieve comparable levels of explained variance. These findings suggest that the choice of a two-dimensional manifold is not merely for visualization convenience, but is fundamentally supported by the intrinsic properties of the brain connectome data.

Finally, Figure 3 displays the concentrated regions of the SCI, MCI, and ADD groups as defined by the fitted Gaussian distributions. As expected, the distributions exhibit a degree of overlap—most notably between the MCI and SCI curves, which overlap more substantially than the MCI and ADD curves. This pattern aligns with clinical observations, as MCI and SCI cases are typically more difficult to distinguish.

Our analysis focuses on the MCI group, since the model was trained specifically on the ADD versus SCI classification task, and minimal overlap is expected between these two groups. In contrast, MCI samples were projected onto the manifold post hoc and were not used during training. To quantify how these projections align with the diagnostic structure, we adopt a Naive Bayes classification approach based on the fitted Gaussian distributions. Under this framework, 27.78% of MCI samples are misclassified as SCI, and 7.03% as ADD, indicating that MCI projections predominantly overlap with the SCI region, which is consistent with their clinical ambiguity.

### 5.4. Neurological Perspective

Based on the manifold regions delineated in Figure 2a and Figure 3, patient cohorts corresponding to SCI, MCI, and ADD were identified. To establish a normative reference, a representative structural network (sNet) for the SCI group was constructed by averaging the edge weights across all sNets within the SCI-defined (red) region. Each individual sNet from the MCI and ADD groups was then compared against this aggregated SCI template.

For each subject in the MCI and ADD groups, deviations in connectivity patterns were assessed by computing the likelihood that each edge weight differed significantly from the Gaussian distribution fitted to the corresponding edge in the SCI group. These per-edge probabilities were transformed via logarithmic accumulation to generate a composite difference score for each cortical region, reflecting its degree of structural divergence from the normative SCI profile.

**Figure 4:**
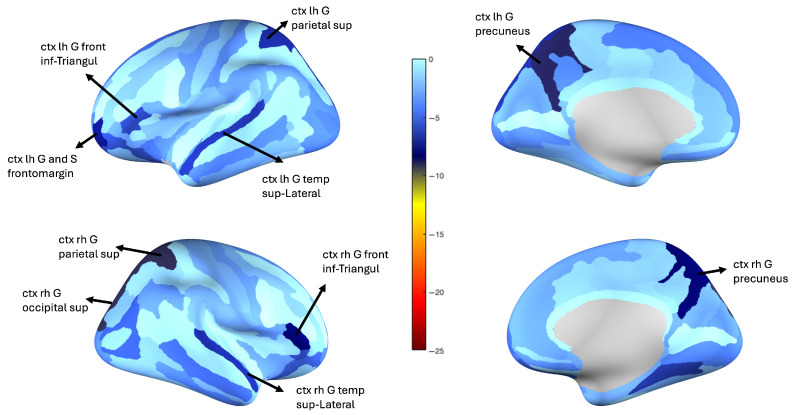
Cortical surface maps showing regional difference scores between the SCI and MCI groups. Scores reflect the logarithmic accumulation of edge-wise deviations from the SCI reference connectome, projected onto the cortex. Warmer colors indicate greater divergence from the normative SCI profile. Notable regions include the right superior parietal lobule, precuneus, occipital cortex, and inferior frontal gyrus.

**Figure 5:**
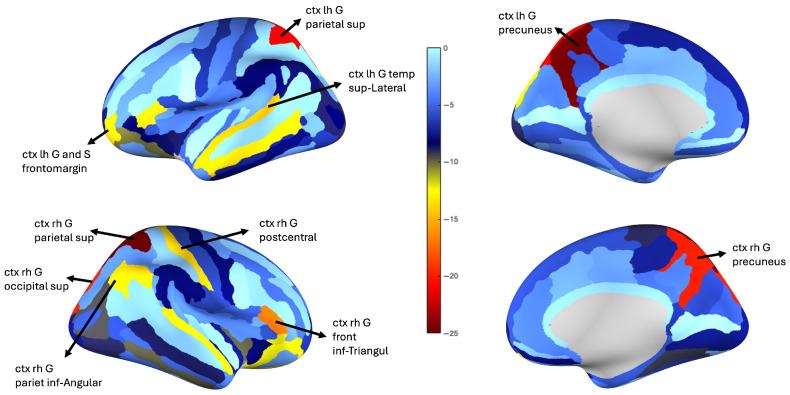
Cortical surface maps illustrating structural divergence between the SCI and ADD groups. Compared to SCI, ADD patients show pronounced disruptions in the bilateral precuneus, superior parietal lobule, postcentral gyrus, and angular gyrus.

The resulting regional difference scores were then visualized by mapping them onto cortical surfaces. This allowed for spatial interpretation of structural alterations across disease stages. These visualizations are presented in Figure 4 and Figure 5, corresponding, respectively, to the SCI–ADD and SCI–MCI comparisons.

A preliminary examination of the structural subnetworks in Figure 4 and Figure 5 reveals distinct patterns of regional vulnerability across the cognitive decline continuum from Subjective Cognitive Impairment (SCI) to Alzheimer’s Disease and Dementia (ADD). The bilateral precuneus, superior parietal lobule, right angular gyrus, and right postcentral gyrus emerge as consistently disrupted regions, each reflecting the breakdown of large-scale brain networks associated with attention, memory, and sensory integration.

The precuneus shows prominent alterations in both SCI–MCI and SCI–ADD comparisons, suggesting its vulnerability in early stages of Alzheimer’s Disease, while medial temporal regions, such as the entorhinal cortex and hippocampus, are typically the earliest sites of amyloid accumulation and neurofibrillary changes, the precuneus may be affected functionally or structurally at a relatively early stage due to its central role within the default mode network (DMN) [61]. Positioned at the intersection of multiple cortical pathways, it has been repeatedly implicated in network-level disruptions observed during the preclinical and prodromal phases. Its bilateral involvement across stages in this analysis supports its potential utility as a sensitive biomarker of large-scale network degeneration [62].

The bilateral superior parietal lobule also demonstrates significant structural alterations in the transition from SCI to ADD. As core components of the dorsal attention network (DAN), these regions are responsible for spatial orientation, attentional reallocation, and working memory [63]. Their alteration may help explain early attentional disturbances observed in individuals at risk for Alzheimer’s, possibly before memory impairment becomes prominent [64,65,66].

Structural changes were observed during the SCI–ADD transition in regions usually spared until the later stages of Alzheimer’s pathology. Unexpected alterations were observed in the right postcentral gyrus (primary somatosensory cortex), bilateral superior occipital gyri (visual network), left frontomarginal gyrus and sulcus (frontoparietal control network), and bilateral superior lateral temporal gyri (somatomotor network), as well as in the triangular part of inferior frontal gyrus and lateral temporal cortex across the SCI–MCI and SCI–ADD comparisons [11,31,67]. Although Braak staging predicts damage to sensory, visual, and executive regions only in later stages (V–VI) [64,66], these areas may be affected much earlier. Initially, their connections may strengthen to compensate for dysfunction in parietal and attention hubs, but as the disease progresses toward stages V–VI, this compensation may fail and structural damage may ensue.

The structural alterations observed in this study suggest a posterior-to-anterior pattern of cortical degeneration in early Alzheimer’s Disease, beginning in the precuneus (a key DMN hub) and extending to parietal and frontal association areas. This supports the notion that Alzheimer’s pathology affects widespread networks, not just memory-related regions, consistent with prior models of network-based neurodegeneration [68,69]. These findings highlight the importance of early interventions targeting not only memory circuits, but also attentional and executive networks that may be vulnerable in preclinical stages.

## 6. Conclusions

This study introduces a novel Graph Neural Network (GNN)-based framework to model and visualize the continuum of cognitive decline across Subjective Cognitive Impairment (SCI), Mild Cognitive Impairment (MCI), and Alzheimer’s Disease and Dementia (ADD) using structural brain connectomes. By combining GIN and GAT layers with attention-based pooling, the model produces discriminative graph-level embeddings, which are subsequently projected onto a two-dimensional manifold via Principal Component Analysis (PCA). The resulting latent space reveals a coherent structural trajectory that aligns with clinical expectations, supporting the hypothesis of a gradual and continuous disease progression. This approach offers a compact and interpretable representation of complex brain network changes, providing new opportunities to characterize disease staging in a neurologically meaningful way. In doing so, it addresses a critical need for models that are not only predictive, but which also offer insights into the underlying mechanisms of cognitive decline.

Several factors contribute to the strength of the proposed framework. Central to its effectiveness is its GNN-based architecture, which is particularly well-suited for brain connectome analysis. It is able to capture topologically meaningful patterns, producing a two-dimensional representation that preserves essential variance and supports interpretable staging. The manifold is robust, non-random, and consistent with established clinical patterns, including regional connectivity differences that align with implicated brain areas. Notably, our findings strongly suggest that brain connectome changes along the cognitive decline continuum are governed by a small number of consistent yet complex patterns, which are effectively captured by the learned manifold dimensions. This representation not only offers a principled approach for dementia staging, but also provides a basis for exploring the evolving organization of brain networks in neurodegeneration.

We note two main limitations of the current study. The modest sample size, while suitable for initial exploration, poses challenges for architectural experimentation and constrains the broader representativeness of the findings. In addition, the cross-sectional design of the study limits the ability to track within-subject progression, which is essential for validating longitudinal changes in the learned manifold. Future work should aim to replicate these findings on larger, homogeneous cohorts, preferably with longitudinal data, to improve statistical power and resolution. Furthermore, mapping the principal components of the learned manifold back to specific neuroanatomical changes could offer deeper insights into the biological underpinnings of cognitive decline. Another promising direction lies in developing improved diagnostic and prognostic methods for clinical cognitive decline by leveraging the low-dimensional manifold, with the potential to enable more effective disease monitoring and individualized staging.

## Figures and Tables

**Table 1:** The GNN + MLP architecture was trained for binary classification tasks among Alzheimer’s Disease (ADD), Mild Cognitive Impairment (MCI), and Subjective Cognitive Impairment (SCI). Performance was evaluated using leave-one-out cross-validation, with ROC-AUC values reported for each method and task as averages over ten runs.

Task	ROC-AUC (Mean ± Std)
ADD/MCI	0.81 ± 0.025
ADD/SCI	0.93 ± 0.005
MCI/SCI	0.55 ± 0.048

**Table 2:** Ablation study: Classification ROC-AUC values for different methods and tasks. The first set of rows presents the baseline results for the model depicted in Figure 1, employing GIN, GAT, and attention pooling sequentially. The subsequent sets systematically remove or replace one component at a time and evaluate the model performance on the specified tasks.

Method	Task	ROC-AUC (Mean ± Std)
Baseline Model		
GIN+GAT+Attention Pool	ADD/MCI	0.81 ± 0.025
	ADD/SCI	0.93 ± 0.005
Ablated Models		
GIN+GAT+Sum Pool	ADD/MCI	0.80 ± 0.018
	ADD/SCI	0.90 ± 0.011
GIN+Attention Pool	ADD/MCI	0.79 ± 0.014
	ADD/SCI	0.90 ± 0.007
GCN+GAT+Attention Pool	ADD/MCI	0.62 ± 0.032
	ADD/SCI	0.78 ± 0.012
GAT+Attention Pool	ADD/MCI	0.74 ± 0.010
	ADD/SCI	0.84 ± 0.016

## Data Availability

The data are not publicly available because data sharing requires the funding agency’s (TUBITAK) approval. Please direct your data related inquiries to the project’s PI, Burak Acar.

## Abbreviations

The following abbreviations are used in this manuscript:

ADD	Alzheimer’s Disease and Dementia
AUC	area under curve
BOLD	Blood-Oxygen-Level Dependent
CDR	Clinical Dementia Rating
CFI-S	Cognitive Functions Instrument—Subject form
CFI-SP	Cognitive Functions Instrument—Study Partner form
CI	cue index
CNN	Convolutional Neural Network
DAN	dorsal attention network
DMN	default mode network
DWI	diffusion-weighted imaging
EMCI	Early Mild Cognitive Impairment
EPI	Echo Planar Imaging
FA	fractional anisotropy
FCSRT	Free and Cued Selective Reminding Test
FFE	Fast Field Echo
fMRI	functional magnetic resonance imaging
fNets	functional networks
FOV	Field of View
GAT	Graph Attention Network
GCN	Graph Convolutional Network
GIN	Graph Isomorphism Network
GNN	Graph Neural Network
LLE	locally linear embedding
LOOCV	leave-one-out cross validation
LSTM	Long Short-Term Memory
MCI	Mild Cognitive Impairment
MLP	multi-layer perceptron
MRI	magnetic resonance imaging
NIA-AA	National Institute on Aging and Alzheimer’s Association
PC	Principal Component
PC1	First Principal Component
PCA	Principal Component Analysis
RK4	fourth-order Runge–Kutta
RNN	Recurrent Neural Network
ROC	receiver operating characteristic
rs-fMRI	resting-state functional magnetic resonance imaging
SCI	Subjective Cognitive Impairment
sNets	structural networks
SOB	sum of boxes
TE	Echo Time
TFE	Turbo Field Echo
TFR	total free recall
TR	Repetition Time
UMAP	Uniform Manifold Approximation and Projection
WL	Weisfeiler–Lehman
